# Identification and characterization of relapse-initiating cells in *MLL*-rearranged infant ALL by single-cell transcriptomics

**DOI:** 10.1038/s41375-021-01341-y

**Published:** 2021-07-24

**Authors:** Tito Candelli, Pauline Schneider, Patricia Garrido Castro, Luke A. Jones, Eduard Bodewes, Dedeke Rockx-Brouwer, Rob Pieters, Frank C. P. Holstege, Thanasis Margaritis, Ronald W. Stam

**Affiliations:** Princess Máxima Center for Pediatric Oncology, 3584 CS Utrecht, The Netherlands

**Keywords:** Cancer genomics, Translational research

## Abstract

Infants with *MLL*-rearranged infant acute lymphoblastic leukemia (*MLL-*r iALL) undergo intense therapy to counter a highly aggressive malignancy with survival rates of only 30–40%. The majority of patients initially show therapy response, but in two-thirds of cases the leukemia returns, typically during treatment. The glucocorticoid drug prednisone is established as a major player in the treatment of leukemia and the in vivo response to prednisone monotreatment is currently the best indicator of risk for *MLL*-r iALL. We used two different single-cell RNA sequencing technologies to analyze the expression of a prednisone-dependent signature, derived from an independent study, in diagnostic bone marrow and peripheral blood biopsies. This allowed us to classify individual leukemic cells as either resistant or sensitive to treatment and show that quantification of these two groups can be used to better predict the occurrence of future relapse in individual patients. This work also sheds light on the nature of the therapy-resistant subpopulation of relapse-initiating cells. Leukemic cells associated with high relapse risk are characterized by basal activation of glucocorticoid response, smaller size, and a quiescent gene expression program with cell stemness properties. These results improve current risk stratification and elucidate leukemic therapy-resistant subpopulations at diagnosis.

## Introduction

Acute lymphoblastic leukemia (ALL) in infants (i.e., children < 1 year of age) is frequently driven by chromosomal translocations of the *mixed lineage leukemia* (*MLL* or *KMT2A*) gene, which occur in ~80% of the cases. Translocations of the *MLL* gene on chromosome 11q23 lead to fusions of the N-terminus of *MLL* to the C-terminus of one of many known translocation partner genes. The majority of infant ALL patients carry one of three recurrent types of *MLL* translocations in which the *MLL* gene becomes fused to either *AF4* (aka *AFF1*; 49% of the cases), *ENL* (aka *MLLT1*; 22% of the cases), or *AF9* (aka *MLLT3*; 16% of the cases) [1]. *MLL*-rearranged infant ALL (MLL-r iALL) represents a rare but highly aggressive type of childhood leukemia that is notoriously characterized by chemotherapy resistance and high relapse rates, leading to a very poor prognosis. Regardless of the type of *MLL* translocation, event-free survival (EFS) rates for *MLL-r iALL* patients remain at 30–40% when treated according to the international collaborative INTERFANT treatment protocol [2, 3], whereas cases without *MLL* translocations fare significantly better at 75–80%.

Despite the massive disparity in EFS, the majority (~95%) of *MLL-r iALL* patients seemingly achieve disease remission after induction therapy. In two-thirds of the cases, however, the leukemia reemerges, typically within the first year from diagnosis and while still on treatment, giving rise to an even more chemotherapy-resistant form.

Relapse occurrence in infant ALL is usually fatal and despite advances in the field its mechanism still needs to be elucidated. Currently, one of the best predictors of future relapse occurrence is the response to a 7-day window of prednisone monotherapy administered prior to induction therapy [2, 3]. This suggests that predisposition to the effects of prednisone at diagnosis might play a pivotal role in the development of relapse. Many hypotheses about relapse emergence also involve cellular heterogeneity [4–10] and a high degree of clonal heterogeneity has been observed in *MLL-r iALL* [11, 12].

To shed light on the interplay between sensitivity to prednisone, cell heterogeneity, and relapse occurrence, we decided to exploit the transformative ability of single-cell RNA sequencing (scRNA-seq) to analyze heterogeneous systems [13–18]. This allowed us to accurately predict which patients were at high risk of leukemia relapse, based on scRNA-seq analyses on diagnostic primary *MLL-r iALL* samples. In addition, we were able to characterize the nature of these relapse-predicting cells.

## Methods

### Patient samples

Bone marrow (BM) biopsies and peripheral blood (PB) samples taken at diagnosis were from infants (<1 year of age) with *MLL*-rearranged ALL and treated according to the international collaborative Interfant-99 and Interfant-06 protocols [2, 3]. We did not distinguish between the two protocols as the treatment differences between the two are minimal and no outcome differences were detected [3]. Samples used were from *MLL*-rearranged pro-B infant ALL patients, carrying either of the two most common *MLL* fusion genes, i.e., *MLL*-*AF4* or *MLL*-*ENL* [19], and with cell viability over 65%. Samples were either from patients with at least 7-year relapse-free survival or from patients who experienced relapse within 2 years after diagnosis. Care was taken to spread attributes such as sex and translocation type across the dataset (Table 1). Informed consent was obtained from the parents or legal guardians according to the Helsinki Declaration. BM and PB samples were processed as described [20]. Leukemic blast percentages (Table 1) were determined microscopically using May-Grünwald–Giemsa stained cytospin preparations.

**Table 1:** Overview of the patient samples used in this study, their characteristics, Interfant risk stratification, and number of sequenced cells.

Sample ID BM	1977N	1702N	635N					8010R	6806R	4662R	4483R						
Sample ID PB	1978N	1703N	636N	1443N	1966N	888N	8812N	8011R	6807R		4484R	3595R	6487R	1776R	1175R	2009R	
Translocation	t (4;11)	t (11;19)	t (4;11)	t (4;11)	t (4;11)	t (11;19)	t% (4;11)	t (4;11)	t (4;11)	t (11;19)	t (11;19)	t (4;11)	t (11;19)	t (4;11)	t (4;11)	t (11;19)	
Gender	Female	Male	Male	Female	Female	Female	Female	Female	Male	Female	Male	Male	Female	Female	Female	Female	
Age at diagnosis (months)^1^	6.5	11.1	2.8	10.3	1.9	5.3	10.3	0	11.3	5.3	3.6	3.5	6.3	0.7	6.6	0.0	
Time to relapse (months)^2^								7.0	10.3	4.8	4.6	11.3	13.4	16.4	21.3	4.5	
Risk stratification adjusted to^3^	Medium	Medium	High	Medium	Medium	Medium	Medium	Medium	Medium	High	High	High	Medium	Medium	Medium	High	
Protocol	Interfant-99	Interfant-99	Interfant-99	Interfant-99	Interfant-99	Interfant-99	Interfant-06	Interfant-06	Interfant-06	Interfant-06	Interfant-06	Interfant-99	Interfant-06	Interfant-99	Interfant-99	Interfant-99	
Lymphoblasts (%) SCS sample^4^	92	95	92	70	91	95	100	98	99	98	96	96	95	93	95	96	
Lymphoblasts (%) initial sample^5^	82	93	94	45	90	95	93	83	86	82	95	93	90	84	92	90	
White blood cell counts at diagnosis^6^	291,000	310,000	571,200	69,900	263,000	226,800	201,000	125,000	75,000	487,400	635,300	348,600	221,500	116,100	25,000	416,000	
Count blasts at day 8^7^	102	162	0	623	495	450	99	44	605	1197	782	200	1250	44	255	94500	
Number of cells BM SORT-seq^8^	109	144	149					187	218	111	162						1080
Number of cells PB SORT-seq^9^	188	267	264	410	603	428	641	231	192		260	617	272	272	290	165	5100
Number of cells PB 10x Genomics^10^	4733	2194	4792					3828	4232		4950						24,729
	1. Days after birth divided by 30.																
	2. No value means no relapse for the duration of follow-up (minimally 7 years).																
	3. Risk stratification according to Interfant-06. For MLL-r iALL there is only medium and high risk.																
	4. Lymphoblasts in bone marrow sample used for single-cell sequencing.																
	5. Percentage leukemic blast cells of the initial sample, at diagnosis.																
	6. White blood cell counts per microliter of blood at diagnosis in ×10^9^/L.																
	7. Leukemic blast cell count per microliter of blood at day 8.																
	8. Number of analyzed leukemic blast cells of bone marrow samples in SORT-seq.																
	9. Number of analyzed leukemic blast cells of peripheral blood samples in SORT-seq.																
	10. Number of analyzed leukemic blast cells of peripheral blood samples in 10x Genomics.																
	Good prognostic factors: Age at diagnosis > 6 months; white blood cell counts at diagnosis < 300 × 10^9^/L; leukemic blast cell count per microliter of blood at day 8 < 1000.																
	Poor prognostic factors: Age at diagnosis < 6 months; white blood cell counts at diagnosis > 300 × 10^9^/L; leukemic blast cell count per microliter of blood at day 8 > 1000.																
Interfant-99	Standard risk (SR): good PRED response, leukemic blast cell count per microliter of blood at day 8 < 1000.																
	High risk (HR): poor PRED response, leukemic blast cell count per microliter of blood at day 8 < 1000.																
Interfant-06	Low risk (LR): *KMT2A* germline.																
	High risk (HR): presence of a KMT2A-rearrangement and age < 6 months at diagnosis and with WBC count > 300 × 10^9^/L at diagnosis or a poor prednisone response.																
	Medium risk (MR): comprising all other KMT2A-rearranged patients.																

### Single-cell RNA sequencing

Samples were sorted into 384-well plates (SORT-seq, primers shown in Supplementary Table 1) or tubes (10x Genomics) using FACS sorting. The gating strategy employed for sorting is shown in Supplementary Fig. 1. See Supplementary Methods for more details.

For SORT-seq, 384-well plates with sorted cells were processed into Illumina sequencing libraries as described [21, 22] and preprocessed as in ref. [23]. Because of their high variation in gene expression, at this stage mitochondrial genes were removed. A minimum transcripts threshold was set to 500 transcripts per cell. The number of detected genes and adequacy of sequencing were evaluated in Supplementary Fig. 2a, b.

10x Genomics processed samples were prepared and sequenced according to the manufacturer’s protocol using the Illumina NextSeq500 sequencer. Reads were processed with the zUMIs pipeline version 2.2.0 using the same genome and annotation version as in ref. [23]. At this stage, mitochondrial genes were removed and all barcodes with less than 500 transcripts were excluded.

### scRNA-seq analysis

**Fig. 1:**
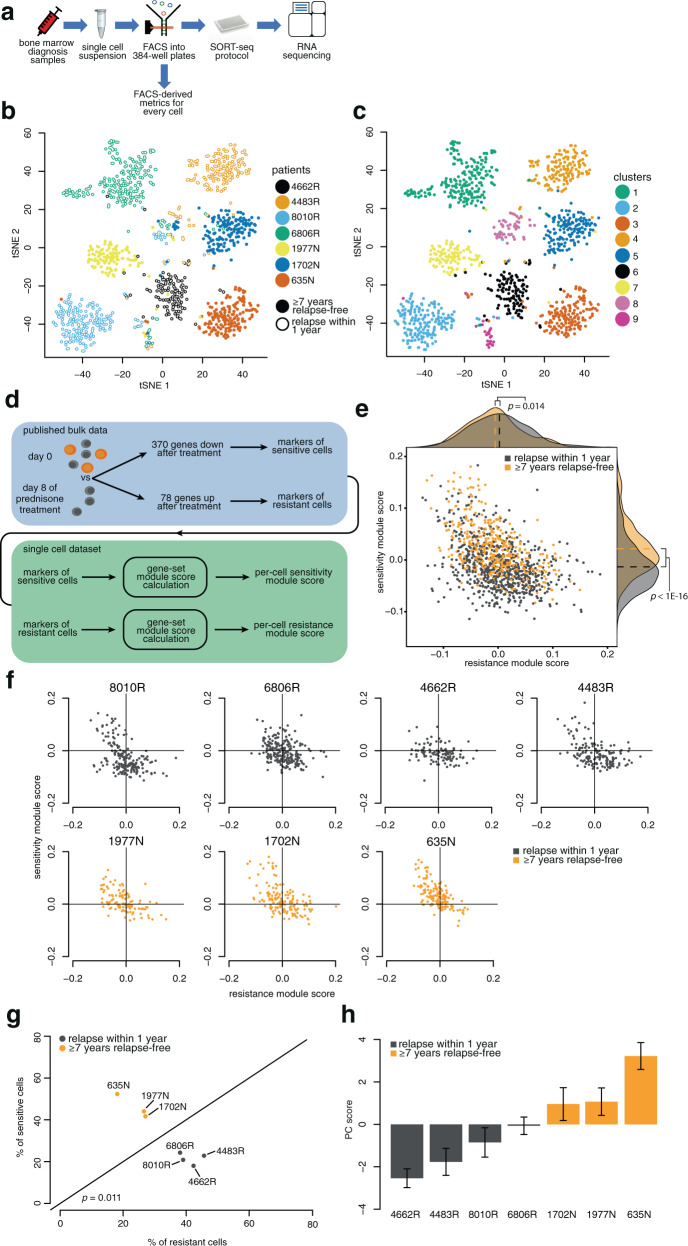
Single-cell drug-sensitivity classification leads to relapse prediction. **a** Experiment design. **b** t-distributed stochastic neighbor embedding (t-SNE) plot of cells labeled according to sample ID, with R indicating patients who suffered relapse and N indicating no relapse. **c** Louvain clustering^24^ projected onto the t-SNE plot. **d** Previously published differential expression data obtained comparing naive and prednisone-treated samples^26^ were applied as gene modules to classify cells for sensitivity (downregulated genes) and resistance (upregulated genes). **e** Gene module scores (*x*- and *y*-axis) for each cell, with cells from patients who later developed relapse labeled gray and cells from relapse-free patients labeled orange. **f** Gene module scores for cells from each patient individually. Cells in the upper-left quadrant are predicted to be more sensitive and in the bottom-right more resistant to treatment. **g** Quantification of the fraction of cells from each patient (from **f**) predicted to be sensitive (*y*-axis) or resistant (*x*-axis). **h** First principal component (PC) calculated using the union of sensitive/resistance module genes for each cell. Bar height represents the mean score per patient. Error bars represent standard error of the mean.

For BM samples, further analysis was performed using R version 3.3.4 and the package Seurat [24] version 2.1.0 with default parameters unless stated otherwise. Per-cell transcript counts were normalized to 3500 transcripts. The first 15 principal components (PCs) of a PC analysis (PCA) were used to generate t-distributed stochastic neighbor embedding plots (Fig. 1b, c, Supplementary Fig. 2) and perform Louvain clustering [24] (Fig. 1c) using a resolution of 1.1. Cluster number 9 consisted of T cells (Supplementary Fig. 2c, d) and was excluded from further analyses.

For PB samples, further analysis was performed using R version 3.6.0 and the package Seurat version 3.0.2 with default parameters unless stated otherwise. Normalization was performed using SCTransform [25]. Gene filtering was performed as in ref. [23]. The following genes were removed from all analyses: XIST and TSIX genes as well as all genes on the Y chromosome and hemoglobin genes. The first 30 PCs were used to perform Louvain clustering [24] using a resolution of 1.

### Gene module scores

Genes used for the calculation of the sensitivity and resistance module scores were obtained from ref. [26] and are shown in Supplementary Table 2. Calculation of module scores was performed using the Seurat AddModuleScore function with modifications [24]. Briefly, each gene is classified into an expression bin according to its average expression across all cells. To obtain the score, for each cell, each chosen gene’s expression is compared to the average of 100 randomly chosen genes from the same expression bin as a control. The difference between each chosen gene’s expression and its matching control value is then averaged across all chosen genes, yielding the final module score.

### Categorization of sensitive and resistant cells

Cells were categorized as sensitive when their sensitivity score was above the median sensitivity score of the complete dataset and their resistance score was below the median resistance score calculated over the whole dataset. Vice versa, cells were categorized as resistant when their resistance score was above and their sensitivity score was below the corresponding median scores of the dataset.

### PC score

The PC score constitutes the first PC of a PCA calculated using the union of sensitivity and resistance module genes on scaled log normalized expression values (see “Gene module scores” section above). As depicted in Supplementary Fig. 3, a high PC score corresponds to cells with a predicted high sensitivity to treatment.

### In vitro prednisolone treatment

In vitro drug exposures were performed by incubation with 100 µg/mL prednisolone (BUFA, Uitgeest, The Netherlands), the liver-activated form of prednisone, or with vehicle for 3 days. Cells were viably frozen [20] and later thawed for scRNA-seq. All processed samples had at least 90% blasts.

### PB differential expression

To determine genes differentially expressed between sensitive and resistant cells in 15 PB samples processed with SORT-seq, we defined cells as sensitive or resistant depending on their module scores (see the “Methods” section). This yielded 1722 cells in each group. Differential expression was calculated using the FindMarkers function with default arguments. The resulting *p* values were Bonferroni multiple-testing corrected. Genes with an adjusted *p* value lower than 0.05 and with an average log fold change (natural log) above 0.20 were considered differentially expressed.

### Gene Ontology (GO) enrichment

GO category enrichment was calculated using the compareCluster function from the clusterProfiler R package [27], see Supplementary Methods for details.

## Results

### Clustering of leukemic cells according to individual patients

To identify subpopulations of cells potentially associated with relapse, we analyzed leukemic cells derived from BM biopsies taken at diagnosis. These samples were obtained from seven *MLL-r iALL* patients covering the two most recurrent *MLL* translocations, t(4;11) and t(11;19), giving rise to the *MLL* fusion genes *MLL-AF4* and *MLL-ENL*, respectively [1–3, 21]. We processed the samples into scRNA-seq libraries using SORT-seq [21] (Fig. 1a), a medium-throughput platform that provides high sensitivity [28] and cytometric data on individual cells. As anticipated, cells clustered largely according to individual patients (Fig. 1b, c). This agrees well with the personalized nature of cancer [29] and the substantial patient-to-patient heterogeneity of *MLL-r iALL* [11, 12].

We identified two clusters with contribution from multiple patients. These were revealed to be highly proliferating blasts (Supplementary Fig. 2c, f–g) and healthy T cells (Supplementary Fig. 2c–e). The latter were removed from further analyses.

Unsupervised clustering did not group leukemic cells by characteristics such as sex, translocation type, or relapse occurrence (Supplementary Fig. 2h), underscoring the distinct nature of individual cancers and the challenge of accurately predicting treatment outcome.

### Single-cell analysis predicts relapse occurrence in *MLL-r iALL* BM biopsies

The glucocorticoid drug prednisone is one of the cornerstones of the treatment of ALLs [30]. The response to 1 week of prednisone monotherapy is considered a major parameter for current risk stratification and a strong predictor of clinical outcome [2, 3, 30]. The response to this drug has been studied by a variety of approaches, including bulk mRNA measurements in samples derived from pediatric ALL patients [26].

Rather than interpreting these results as revealing a prednisone gene expression response, we reasoned that apparent up- and downregulation of specific genes might be at least partially driven by a process of Darwinian selection. Gene signatures specific to a preexisting subset of prednisone-resistant cells would emerge as upregulated after treatment by virtue of their higher survival rate even if their expression levels remain constant, while signatures specific to cells sensitive to treatment would appear downregulated for the opposite reason (Fig. 1d). Following this logic, genes upregulated after prednisone exposure mark leukemic cells with a high chance of surviving treatment, while genes with apparent downregulation represent markers of cells sensitive to treatment and therefore preferentially eliminated by prednisone exposure.

To explore this possibility, we took advantage of published differential expression results from the work of Rhein et al. [26] obtained by comparing prednisone-treated samples with matched diagnosis samples. We considered two gene modules consisting of 78 upregulated and 370 downregulated genes (Supplementary Table 2) [26], respectively. Based on the expression of the two gene modules, we classified individual cells as being sensitive or resistant to therapy. The distribution of cells is a continuum from apparent sensitivity to apparent resistance and the two modules strongly anticorrelate with each other (Fig. 1e). This strengthens the notion that these are not two independent signatures, but a common set of intrinsic properties that are mutually exclusive. Strikingly, labeling the cells according to future relapse occurrence reveals a significant difference in both modules, implicating the sensitivity and resistance markers in the process of relapse development.

To further test the predictive capability of our data, we examined the single-cell classification in individual patients (Fig. 1f). Visual inspection indicates more resistant-predicted cells (Fig. 1f, bottom-right quadrants) in patients who eventually relapsed and more sensitive-predicted cells (upper-left quadrants) in patients who remained relapse-free. For quantitative comparison, we calculated the percentage of cells classified as sensitive/resistant for each diagnostic sample. This yielded a strong distinction between patients with and without relapse (Fig. 1g). As a further control and for future ease of comparison with other metrics, we used PCA to assign a singular value to each cell representing the position along the sensitivity-resistance continuum (Fig. 1h, see Supplementary Fig. 3a–d for how well the first PC embodies the signal from the two modules). As expected, PC score is able to differentiate between long-term survivors and relapsing patients. Treatment resistance is an obvious determinant of outcome [31] and taken together, these analyses suggest that such property might already be detectable at diagnosis, possibly owing to a preexisting subpopulation of resistant cells.

### In vitro prednisolone treatment enriches for cells classified as resistant

**Fig. 2:**
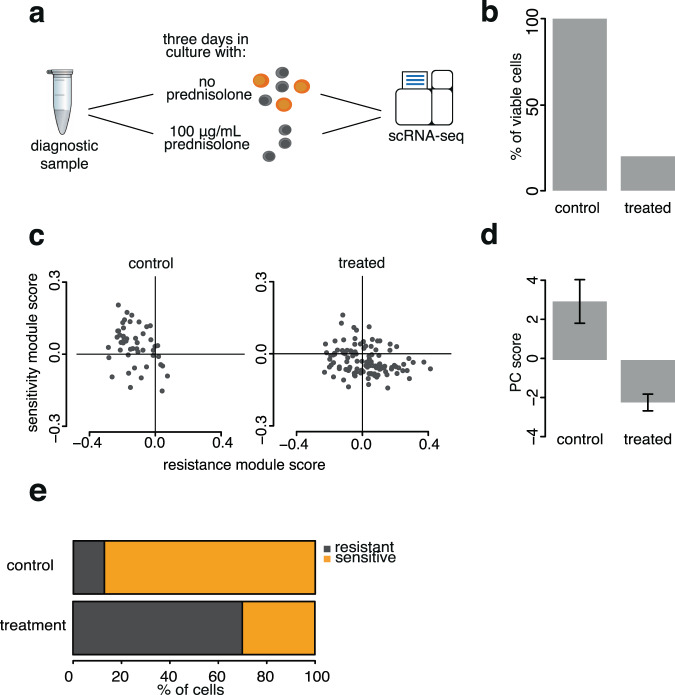
In vitro treatment enriches for cells classified as resistant. **a** Untreated leukemic cells from bone marrow diagnostic biopsy were cultured with and without prednisolone. **b** Cell viability after treatment. **c** scRNA-seq-based sensitivity and resistance module scores of viable cells from control and treated cultures as in Fig. 1f. **d** First PC score as in Fig. 1h. **e** Fractions of cells classified as sensitive/resistant in control and treated samples.

The single-cell relapse prediction is based on the idea that gene expression response to prednisone [26] reflects survival of treatment-resistant cells (Fig. 1d). To further test this, untreated leukemic cells from a diagnosis sample were exposed to prednisolone (the liver-activated form of prednisone) in vitro (Fig. 2a). As expected, treated cells are less viable (Fig. 2b), consistent with prednisolone activity. Single-cell classification shows that leukemic cells predicted to be resistant are present in a lower proportion in the control sample and become highly enriched after elimination of the sensitive cells by prednisolone (Fig. 2c–e). This agrees with our interpretation that the previously published prednisone response genes are indeed markers for treatment sensitivity/resistance (Fig. 1d) and is consistent with the two programs been present in the samples before any treatment.

### Relapse prediction is robust across scRNA-seq technologies and leukemic niches

Encouraged by our findings in a relatively small cohort of primary *MLL-r iALL* BM biopsies, we repeated our analysis on PB samples. This allowed us to greatly increase the number of patients included in this study, and validation of these results in PB could open more avenues for future clinical applications.

In addition, to further validate our findings, we evaluated our PB results using two different techniques, SORT-seq and the industry standard 10x Genomics.

**Fig. 3:**
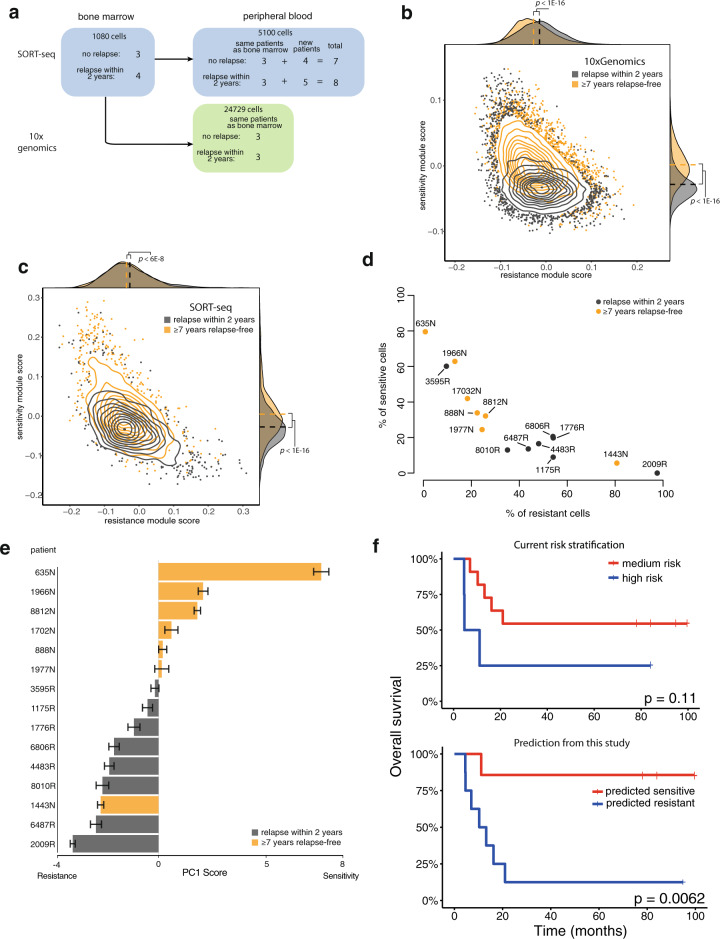
Relapse prediction is confirmed in an expanded cohort of 15 peripheral blood samples. **a** Experimental design. **b** Gene module scores distribution for all cells processed with 10x Genomics. Cells from patients who later developed relapse labeled gray and cells from relapse-free patients labeled orange. **c** As **b**, but for cells processed with SORT-seq. **d** Quantification of the fraction of cells from each patient predicted to be sensitive or resistant. **e** Barplot showing the average PC score for each patient. Error bars represent standard error of the mean. **f** Kaplan–Meier plots showing the performance of current risk stratification versus the classification of this study.

As an initial pilot we used matched PB samples corresponding to six of the BM samples analyzed above (Table 1, Fig. 3a), and processed them with both SORT-seq and 10x Genomics. After exclusion of healthy cells from the analysis (Supplementary Fig. 4a), we again detected differences in the expression of the sensitivity and resistance module between long-term survivors and relapsing patients in both technologies (Fig. 3b, c), consistent with previous results.

Relapse status classification of these six PB samples was also consistent with earlier findings in BM (10x Genomics: Supplementary Fig. 4b, c, SORT-seq: together with additional samples in Fig. 3d, e, Supplementary Fig. 4d, e) and did not depend on the technology despite the difference in number of analyzed cells (Fig. 3a). Taken together, these results confirm that our classification signature is robust both across scRNA-seq technologies and across leukemic niches (PB and BM), further validating the general applicability of these findings.

### Relapse prediction in an extended cohort of *MLL-r iALL* PB samples

We performed SORT-seq on nine additional primary *MLL-r iALL* PB samples taken at diagnosis (Supplementary Fig. 5a–c), resulting in an extended cohort comprising of seven patients who remained relapse-free for at least 7 years, and eight patients who relapsed within the first 2 years from diagnosis. Focusing on this extended cohort, we again asked whether the percentage of therapy-resistant and -sensitive cells present at diagnosis could be indicative of future relapse. Despite 2 out of the 15 samples being misclassified (a long-term survivor and an early relapsing patient), we observed a strong association between the proportion of resistant cells at diagnosis and relapse occurrence (Fig. 3d, e, Supplementary Fig. 5c). Taken together, these results show that higher proportion of drug-resistant cells in PB blasts strongly correlate with relapse occurrence in an extended cohort of 15 infants with *MLL*-rearranged ALL. Relapse prediction based on this extended dataset is still overall superior to current risk stratification (Fig. 3f). Interestingly, current metrics used for risk stratification perform as well as this study when evaluating long-term survivors (6/7 correct predictions in both cases) but fall substantially short when evaluating patients who eventually relapse (3/8 correct predictions, compared to 7/8 correct predictions in this study). This difference highlights the need for improved risk assessment, especially for patients that are most at risk.

### Characterization of relapse-initiating leukemic cells identified by single-cell analysis

**Fig. 4:**
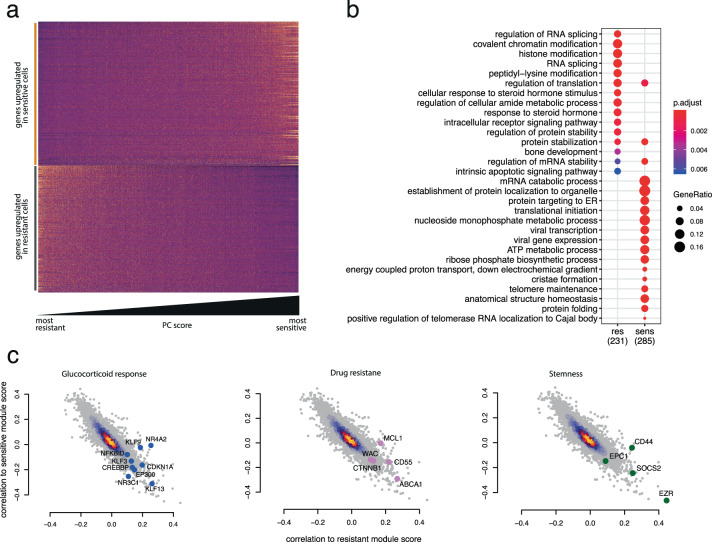
Cells associated with high relapse risk are quiescent and show activated prednisone response. **a** Expression heatmap of all differentially expressed genes between cells classified as sensitive and resistant. cells (columns) are ordered by PC score, reflecting a gradient from resistant to sensitive. **b** Gene Ontology categories enriched in the markers of sensitive and resistant cells. Gene ratio represents the fraction of differentially expressed genes in each category. **c** Spearman correlation of all genes with either sensitivity (*y*-axis) or resistance (*x*-axis) module score. Each plot is the same, but different categories of genes are highlighted in each plot.

To further characterize sensitive/resistant cells, we first compared them by differential expression analysis (PB: Supplementary Table 3, BM: Supplementary Table 4) and then performed GO enrichment on the resulting markers. As detected for the module scores themselves (Figs. 1e and 3b, c), sensitivity and resistance markers are also expressed as a continuum of characteristics rather than distinct subtypes in both PB (Fig. 4a) and BM (Supplementary Fig. 5d). GO enrichment indicates that cells with predicted higher sensitivity to treatment are metabolically more active (Fig. 4b). This resonates with our findings in BM samples, which revealed that sensitive cells are actively proliferating (Supplementary Fig. 5e, f). The anticorrelation of sensitivity and resistance markers expression also emphasizes the converse trend: resistant cells are associated with reduced metabolic (Fig. 4b) and cell-cycle activity (Supplementary Fig. 5e, f) and appear to represent more quiescent or dormant cells. Therefore, we asked whether resistant cells would appear smaller by virtue of their quiescence and lack of metabolic activity. This trend was consistently observed in BM samples using FACS forward scatter as a proxy for cell size (Supplementary Fig. 6a, b). However, further size analysis by both microscopy and FACS on a patient-by-patient basis—while highlighting a significant global trend in both PB and BM when patients were aggregated according to future relapse occurrence (Supplementary Fig. 6d–f, aggregate)—was not able to stratify patients as accurately as our gene signature (Supplementary Fig. 6c–f).

While quiescence/activity seems to be an important dichotomy characterizing the two ends of the resistance/sensitivity continuum, several interesting GO categories also appear differentially enriched (Fig. 4b). Notably, categories comprising steroid hormone response and apoptotic signaling pathway suggest intrinsic differences in the regulation of these processes and might explain the differential treatment sensitivity. In order to relate the expression levels of these genes to the sensitivity/resistance modules that associate with relapse occurrence, we correlated the expression of all genes with said module scores and represented the results as scatterplots (Fig. 4c).

We identified several groups of genes with high correlation to the resistance module score, relating to glucocorticoid response, drug resistance, and cell stemness. In the first group, we found *NR3C1*, the gene encoding the glucocorticoid receptor, as well as several of its downstream targets such as the *KLF* family of genes, *CDKN1A*, and *CREBBP* [32–34]. This suggests that therapy-resistant cells already exhibit at least a partially activated glucocorticoid response before treatment and we speculate that this may blunt the effects of subsequent prednisone administrations. We identified several additional genes that may contribute to the survival of resistant cells by mediating drug resistance. *CTNNB1* [35] and *MCL1* [36, 37] have both been previously implicated in establishing drug resistance in *MLL*-driven leukemic cells and additional death escape mechanisms might be provided by the efflux transporter *ABCA1* and antiapoptotic activity of *CD55* [38].

A number of stemness markers such as *CD44, EPC1, SET2D*, and *SOCS2* seemed to correlate very well with our resistance module score and may explain how these cells are able to avoid apoptosis while maintaining replicative potential. In particular, *EPC1* has been reported to sustain the oncogenic potential of the leukemic stem cells in *MLL*-rearranged acute myeloid leukemia [39] and *SET2D* has been recently implicated in safeguarding the genomic integrity of *MLL*-rearranged leukemias [40]. Expression of these factors might provide an *MLL*-rearrangement-specific contribution to the resilience of resistant cells.

Taken together, these results point at a continuum of characteristics present in treatment-naive samples as a determining factor of relapse occurrence, highlighting the role of quiescence, unstimulated glucocorticoid response activation, and apoptosis escape mechanisms.

### Lower amounts of transcripts in relapse-associated cells hampers classification by bulk mRNA expression analyses

Beyond indicating the cells from which relapse arises and the potential for improving treatment of these vulnerable patients, this study also reveals why single-cell analyses may in some cases outperform bulk mRNA approaches for patient classification. The smaller cells associated with higher risk of disease relapse have substantially lower numbers of transcripts (Supplementary Fig. 7a). This fits with quiescence/dormancy as a means to escape chemotherapy and means that bulk mRNA data will not proportionately represent the relative abundance of such cells. Indeed, applying the gene modules (Fig. 1) on previously published bulk mRNA *MLL-r iALL* datasets [12, 41, 42] does not result in a relapse/non-relapse distinction (Supplementary Fig. 7b). Bulkifying the BM scRNA-seq data by complete pooling of all transcripts yields a dataset that also does not discriminate well (Supplementary Fig. 7c). However, pooling the scRNA-seq data after downsampling so that each cell contributes an equal number of transcripts does yield “bulk” data on which the modules discriminate between patients who do and do not relapse (Supplementary Fig. 7d).

## Discussion

To date, *MLL-*r iALL remains an aggressive and difficult-to-treat childhood malignancy. Although induction therapy leads to complete remissions in the vast majority of cases (~95%), two-thirds of the patients experience disease relapse within 1 year from diagnosis, while treatment is still ongoing [2, 3]. This suggests that most of the blasts are responsive to treatment, while a small subpopulation of therapy-refractory cells survives to initiate relapse. In this study, we performed scRNA-seq on 15 diagnosis samples from patients with *MLL*-r iALL. We then used an independently generated gene signature to predict future relapse occurrence correctly in 13 out of the 15 cases, substantially improving on the performance of current risk stratification. In addition, we characterized the subpopulation of therapy-refractory cells, finding them associated with small size, quiescent nature, and heightened glucocorticoid response. Clinical outcome seems to be largely correlated with the abundance of such therapy-resistant leukemic cells. Their detection and further characterization have tremendous potential to drastically improve risk stratification and guide the development of new drugs [11, 12].

Current risk stratification of *MLL-r iALL* involves categorizing patients into either being medium risk or high risk, based on age at diagnosis, white blood cell counts, and the in vivo response to 7 days of prednisone treatment. Although this division does lead to significant differences in clinical outcome (Fig. 3f) [2, 3], it is still often inaccurate, especially for patients that have a high risk of relapse. A possible explanation for this may lie in some of the criteria by which patients are currently being categorized. For instance, one of the most important criteria for risk stratification is COunt of BLAsts at day 8 (COBLA8), representing the count of surviving blasts after 7 days of prednisone monotreatment. Although this measurement is certainly associated with future relapse occurrence, it is often inaccurate and possibly influenced by confounding factors such as differences in initial WBC. In our scRNA-seq-based relapse-prediction model, we improved upon the predictive power of COBLA8 by analyzing the gene expression patterns that characterize surviving cells and finding this signature back in naive untreated diagnostic samples. This allowed us to classify cells as either sensitive or resistant to treatment and to show that the relative proportion of resistant cells in a sample is strongly correlated with relapse occurrence. The direct correlation between expression of the resistance signature and treatment outcome suggests that the signature represents general resistance to chemotherapeutics rather than being specific to prednisone. This is not surprising given the well-known association between COBLA8 and relapse occurrence, but it does raise the question of how a prednisone-associated gene expression pattern is able to affect general therapy resistance.

In our analyses, we found that an activity-quiescence continuum is the most prominent feature separating resistant cells from sensitive cells. Although unlikely to be directly associated with prednisone, it reflects the well-documented resilience of quiescent cells to chemotherapy and suggests that the resistance signature might represent not only prednisone resistance but also multiple therapy-escape mechanisms. This view is further supported by several classes of genes we found enriched in resistant cells. Detection of general mediators of drug resistance and efflux transporters argues for broad therapy resistance, while stemness markers typical of leukemic stem cells might help escape drug-induced cell death and maintain replicative potential. Taken together, these results argue for a model where prednisone monotreatment selects for cells that are small, quiescent, and generally resistant to chemotherapy, setting the stage for future research to characterize them more in depth and decode their therapy-resistance mechanisms.

There are several aspects and limitations of this study that will need to be addressed in order to help translate this knowledge to the clinic. scRNA-seq is not yet a routine lab technique and application of bulk RNA-seq to detect the gene signature suffers from quantification problems owing to the smaller RNA content of resistant cells. Identification of an easily detectable hallmark could help offset this problem and simplify the quantification of resistant cells. However, investigation of clonality and mutation analysis might be required to identify DNA-based hallmarks that are not affected by the smaller size of resistant cells. Despite considerable success, two patients in the cohort were misclassified by our method. At this stage we cannot exclude that specific mutations might act as epistatic factors, bypassing the drug escape mechanisms and resulting in relapse development. Finally, validation of this signature on vast numbers of patients—while essential for inclusion in upcoming trials—is problematic both due to the technique and to the rarity of the disease.

Taken together, these results demonstrate how single-cell sequencing can be used to further our understanding of cancer cell population dynamics and use them for accurate risk assessment. Eventually, elimination of these therapy-resistant cells during early phases of the treatment may well prevent relapse occurrence in a substantial number of cases, leading to increased survival.

## Supplementary information

Supplementary methods

Supplementary Table 1

Supplementary Table 2

Supplementary Table 3

Supplementary Table 4

Supplementary Figure 1

Supplementary Figure 2

Supplementary Figure 3

Supplementary Figure 4

Supplementary Figure 5

Supplementary Figure 6

Supplementary Figure 7

Supplementary figure legends

## Data Availability

Datasets generated for this study have been deposited in EGA under accession number EGAS00001003986 and are available upon approval by the Princess Máxima Data Access Committee.

## Code availability

Any custom code used in this study is available upon request.
