# Supplementary material for: Identification and characterization of relapse-initiating cells in MLL-rearranged infant ALL by single-cell transcriptomics
Source: Leukemia. 2021 Jul 24;36(1):58–67. doi: 10.1038/s41375-021-01341-y (PMC8727302; doi:10.1038/s41375-021-01341-y)
Supplement: Supplementary file 1 — Supplementary methods [file 41375_2021_1341_MOESM1_ESM.docx]

**Supplementary methods**

Identification and characterization of relapse-initiating cells in MLL-rearranged infant ALL by single cell transcriptomics

Tito Candelli*, Pauline Schneider*, Patricia Garrido Castro, Luke A. Jones, Eduard Bodewes, Dedeke Rockx-Brouwer, Rob Pieters, Frank C.P. Holstege#, Thanasis Margaritis#, Ronald W. Stam#

*Princess Máxima Center for Pediatric Oncology*

**Methods**

**Fluorescence-activated cell sorting (FACS)**

Samples were thawed and resuspended in 1xPBS, 2mM EDTA, 0.5%BSA and a viability stain (2.5 *μg/mL* 7AAD or 5 *μg/mL* DAPI (BD Biosciences, San Jose, CA, USA)) in a concentration of approximately 1 million cells/mL. Viable single cells were sorted based on forward/side scatter properties and 7AAD/DAPI staining using FACS (FACSAria III, BD Biosciences for BM samples; MoFlo Astrios Cell Sorter, Beckman Coulter for PB samples). SORT-seq samples were sorted into 384-well plates (Bio-Rad, Veenendaal, The Netherlands) containing 10 µL mineral oil (Sigma-Aldrich, Zwijndrecht, The Netherlands) and 50 nL of barcoded reverse transcription primers as described (26) (Supplementary Table 1). 10xGenomics samples were sorted into tubes. Data was acquired using FACSDiva version 8.0.1 for the FacsAria sorter or Summit version 6.3.1 for the Astrios sorter, the gating strategy employed for sorting is shown in Supplementary Figure 1.

**FACS metrics**

Index sorting files containing cytometric data for each sorted cell were recovered for BM samples plates. This allowed association between transcriptomic data and forward scatter area values as depicted in Supplementary Figure 6. To obtain forward scatter area values for larger populations of cells, Flow Cytometry Standard (FCS) files for each of the samples were analysed. These samples were gated by the same strategy used for sorting into plates (Supplementary Figure 1), yielding cytometric data on a minimum of 4224 cells (sample 635N) and maximum of 170866 cells (sample 8010R). When comparing forward scatter area values across groups of patients (Supplementary Figure 6d, aggregate) an identical number of cells (n=4224) were taken from each patient to ensure equal representation.

**Module Score Calculation**

Module score calculation was performed with the following modification: when sampling control genes, the same gene can be selected multiple times. This ensures that the distribution of values is centred around zero and eliminates biases due to uneven number of genes when two modules are compared.

**Gene Ontology Enrichment**

Arguments and databases used to calculate gene ontology enrichment were: the org.Hs.eg.db annotation database (OrgDb = “org.Hs.eg.db”, version 3.5.0), the biological process ontology (ont=”BP”), a *p-*value and *q-*value cut-off of 0.01 and 0.05 respectively (pvalueCutoff = 0.01, qvalueCutoff = 0.05) and a minimum gene set size of 15 (minGSSize = 15). Results were subsequently simplified using the simplify function.

**T-B similarity score**

To compute the T-, B-cell similarity score (Supplementary Figure 2d-e), two module scores were calculated as described, using markers of T and B cells respectively. T-cell markers were: CD3E, CD3D, CD3G, CD8A, CD7, CD4, TRBC1. B-cell markers were: CD19, CD79A, MS4A1, CD22. The scores were rescaled so that the values for each module is between 0 and 1 and then the B-cell module score was subtracted from the T-cell module score for each cell. The resulting similarity score ranges between -1 and +1, with the highest values representing high similarity to T-cells and the lowest values high similarity to B-cells.

**Cell-cycle analysis**

Cell-cycle phase (Supplementary Figures 2g, 5f) was determined for each cell using the Seurat (1) (version 2.1.0) CellCycleScoring function with default parameters. The list of marker genes for S and G2M phase are as described (2).

**Survival analysis**

To obtain the Kaplan-Meier curves for current risk stratification and this study’s prediction we used the function “survfit” from the R package “Survival” version 3.2-7 and “ggsurvplot” from the R package “Survminer” version 0.4.8. P-values were calculated using chi-squared test for comparison of categorical variables.

**Bone Marrow Differential expression**

To find markers of the cell clusters depicted in Figure 1c, differentially expressed genes were determined with the FindAllMarkers function in Seurat (1). Differential expression was assessed using the bimodal test (argument test.use = “bimod”, only.pos = TRUE). The resulting *p*-values were Bonferroni multiple-testing corrected. Genes with an adjusted *p*-value lower than 0.05 and with an average log fold-change (natural log) above 0.20 were considered differentially expressed. This resulted in 389 cluster-8 specific genes and 428 cluster-9 specific genes. To determine genes differentially expressed between sensitive and resistant cells (Supplementary Figure 5a), two groups were composed consisting of the 15 cells with the lowest PC score from each patient (resistant cells) and the 15 cells with the highest PC score from each patient (sensitive cells). These two groups were compared using the function FindMarkers with the same arguments as above. To eliminate the effect of patient-specific gene expression, we excluded all genes to which a single patient contributed more than 40% of the total number of cells expressing that gene. Genes exceeding the thresholds described above were considered differentially expressed (Supplementary Table 4).

**removal of healthy cells from Peripheral blood samples**

Clusters detected for both 10x and sort-seq performed on peripheral blood samples were analyzed with SingleR version 1.0.1 with default parameters. clusters classified as pro-B were kept as tumor, while clusters classified as B-cell, monocytes, T-cells, NK cells, or erythroblasts were removed from subsequent analyses.

**Gene correlation with module scores.**

Expression of all genes in the dataset were correlated to the sensitivity and resistance module score using Spearman correlation. The resulting coordinates were plotted in figure 4c.

**Cell size determination by microscopy**

Images of May-Grünwald Giemsa stained cytospin slides were made using the DM200 LED microscope (Leica, Amsterdam, The Netherlands) and utilized to create outlines of the cells to determine cell size using ImageJ software (3). Briefly: RGB colour photos were converted into 32-bit pictures and the “threshold” function with the black and white (B&W) setting was used to outline the cells. The outlines were filled by using the ImageJ features “fill holes”, converted using “convert to mask”, and separated using “watershed”. Small outlines with an area below 120 pixel^2^ were ignored. Finally, only the leukemic blast cells were used for analysis as shown in Supplementary Figure 6c.

**Bulk mRNA data analyses**

The bulk mRNA datasets used for the classifications depicted in Supplementary Figure 7b are DNA microarray(4) and RNA-seq datasets (5, 6) from infant ALL patients at diagnosis. The microarray data was processed as described (4). For the RNA-seq data, paired-end reads were mapped with STAR (7) version 2.6.1 and read assignment was performed with featureCounts 1.6.4 (8), using genome and annotation versions as described in the scRNA-seq section above.

To resemble as close as possible the scRNA-seq analysis, reads were assigned to features according to the hierarchical structure described previously (9). Reads were converted to Transcripts Per Million (TPM) and normalized to 1 million transcripts. Sensitivity and resistance module scores were calculated as described above (Gene module score section) on the matrix of bulk mRNA datasets.

**scRNA-seq bulkification**

For each scRNA-seq dataset, contributions from all cells were pooled by summing all transcripts for each gene. The resulting values were then normalized to 1 million transcripts (Supplementary Figure 7c, top panel). To obtain bulkified scRNA-seq with equal contribution from each cell (Supplementary Figure 7d), the number of transcripts from each cell was down- or up-sampled to 1500 according to the original distribution of transcripts in that cell. Datasets were subsequently bulkified as above. In order to estimate sampling errors, the procedure was repeated 30 times.

**Box plots**

When box plots are used to summarize distributions, the central line represents the median. The lower and upper limits of the box represent the 25^th^ and 75^th^ percentile respectively. The dashed lines extend to 1.5 times the Interquartile Range (IQR) of the distribution. Data points lying beyond this limit are represented as individual dots (outliers). In Supplementary Figure 6d-f these outliers were omitted due to space limitations.

**Statistical tests**

Distributions of sensitivity and resistance module scores in cells from early relapse patients (*n=*678 cells) and long-term relapse-free survivors (*n=*402 cells, Figure 1e) were compared using a two-tailed Welch’s two sample t-test. The same test was used to compare PB early relapse and relapse-free survivors in figure 3b-c (figure b: *n*=719 relapse-free cells, *n*=683 early relapse cells; figure c: *n=*11719 relapse-free cells, *n*=13010 early relapse cells)

The significance of the separation between early relapse and long-term relapse-free survivors in Figure 1g was assessed by first calculating the best linear fit using all points and then projecting them onto the resulting regression line. This ensures that the points are arrayed along a single dimension and that the variance of the overall distribution is maximized. A two-tailed Welch’s two sample t-test was then performed between the two groups containing *n*=3 relapse-free survivors and *n*=4 early relapse patients.

Spearman’s correlation between gene expression and module score in figure 4c and between PC score and forward scatter area in Supplementary Figure 6b was calculated using the R “cor” function using method = “spearman” as an argument. Pearson’s correlation between PC score and sensitivity/resistance module scores (Supplementary Figure 3a-d) were calculated as above but using method = “pearson” as argument. Aggregate distribution depicted in Supplementary Figure 6d-e were compared using two-tailed Welch’s two sample t-test. For Supplementary Figure 6d, *n=*12672 cells for the sensitive category, and *n=*16896 for the resistant category. For Supplementary Figure 6e, *n=*288 cells for the sensitive category and *n=*428 for the resistant category. Survival curves in figure 3f were compared with a log-rank test.

1. Butler A, Hoffman P, Smibert P, Papalexi E, Satija R. Integrating single-cell transcriptomic data across different conditions, technologies, and species. Nat Biotechnol. 2018;36(5):411-20.

2. Tirosh I, Izar B, Prakadan SM, Wadsworth MH, 2nd, Treacy D, Trombetta JJ, et al. Dissecting the multicellular ecosystem of metastatic melanoma by single-cell RNA-seq. Science. 2016;352(6282):189-96.

3. Schneider CA, Rasband WS, Eliceiri KW. NIH Image to ImageJ: 25 years of image analysis. Nat Methods. 2012;9(7):671-5.

4. Stam RW, Schneider P, Hagelstein JA, van der Linden MH, Stumpel DJ, de Menezes RX, et al. Gene expression profiling-based dissection of MLL translocated and MLL germline acute lymphoblastic leukemia in infants. Blood. 2010;115(14):2835-44.

5. Agraz-Doblas A, Bueno C, Bashford-Rogers R, Roy A, Schneider P, Bardini M, et al. Unraveling the cellular origin and clinical prognostic markers of infant B-cell acute lymphoblastic leukemia using genome-wide analysis. Haematologica. 2019;104(6):1176-88.

6. Andersson AK, Ma J, Wang J, Chen X, Gedman AL, Dang J, et al. The landscape of somatic mutations in infant MLL-rearranged acute lymphoblastic leukemias. Nat Genet. 2015;47(4):330-7.

7. Dobin A, Davis CA, Schlesinger F, Drenkow J, Zaleski C, Jha S, et al. STAR: ultrafast universal RNA-seq aligner. Bioinformatics. 2013;29(1):15-21.

8. Liao Y, Smyth GK, Shi W. The Subread aligner: fast, accurate and scalable read mapping by seed-and-vote. Nucleic Acids Res. 2013;41(10):e108.

9. Candelli T, Lijnzaad P, Muraro MJ, Kerstens H, Kemmeren P, van Oudenaarden A, et al. Sharq, A versatile preprocessing and QC pipeline for Single Cell RNA-seq. bioRxiv. 2018:250811.
