# Supplementary figures and images for: Identification and characterization of relapse-initiating cells in MLL-rearranged infant ALL by single-cell transcriptomics
Source: Leukemia. 2021 Jul 24;36(1):58–67. doi: 10.1038/s41375-021-01341-y (PMC8727302; doi:10.1038/s41375-021-01341-y)

a

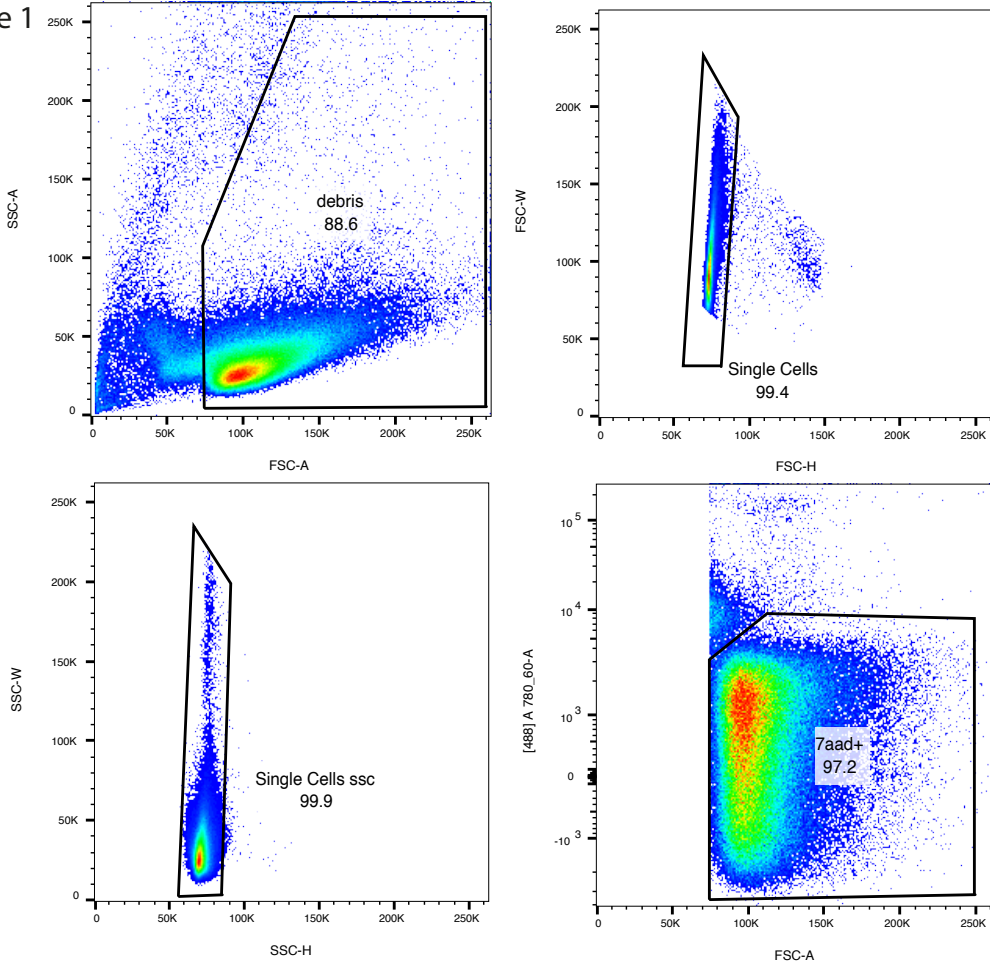

b

PS\_live\_patient\_PB\_InfALL    sort\_1443\_pl1\_PMC678\_3    D:\...\20200703\_PS\_InfALL\_10X\_SortSeq\sort\_1443\_pl1\_PMC678\_3.fcs

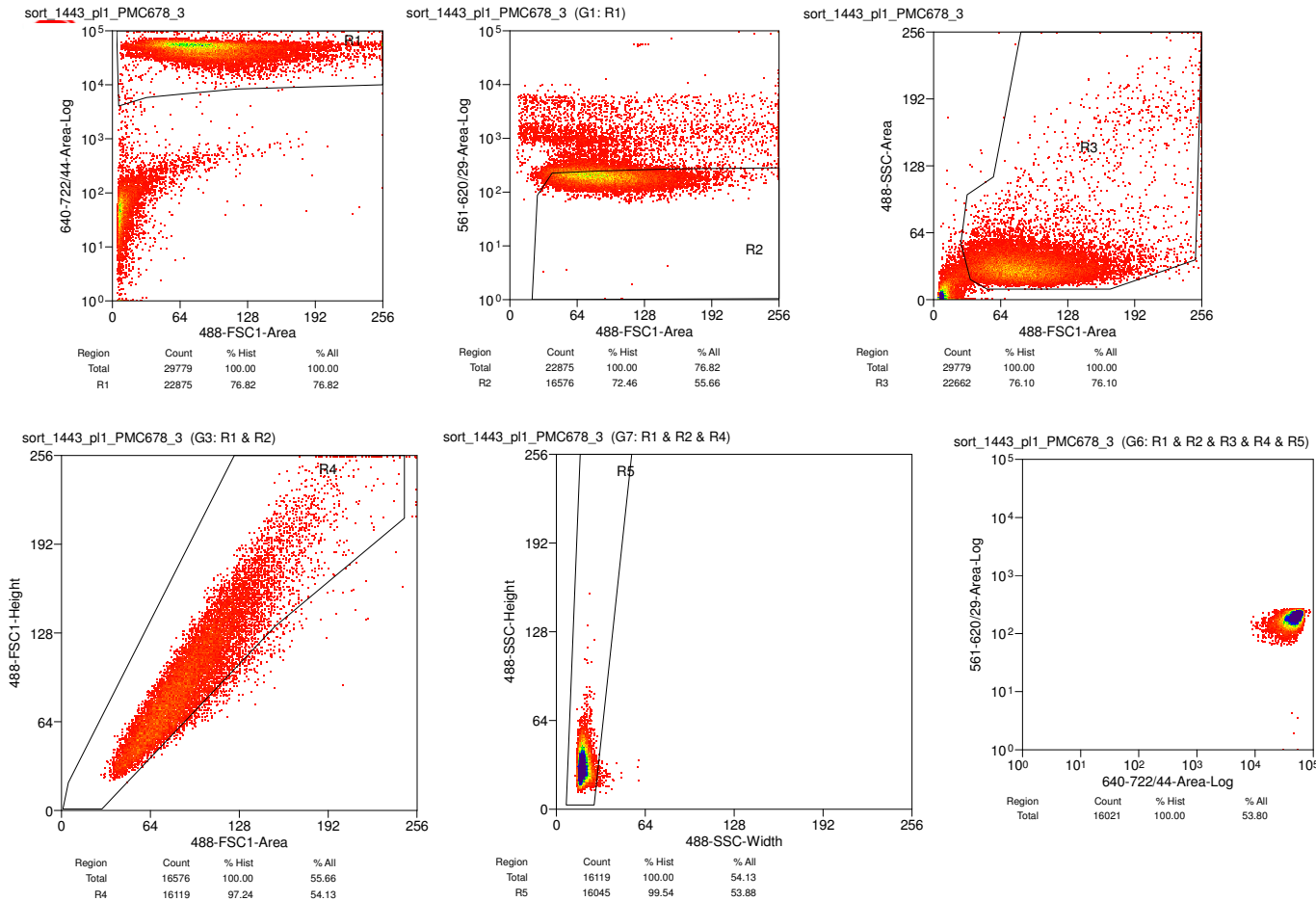

Supplement: Supplementary file 6 — Supplementary Figure 1 [file 41375_2021_1341_MOESM6_ESM.pdf]

Supplementary Figure 2

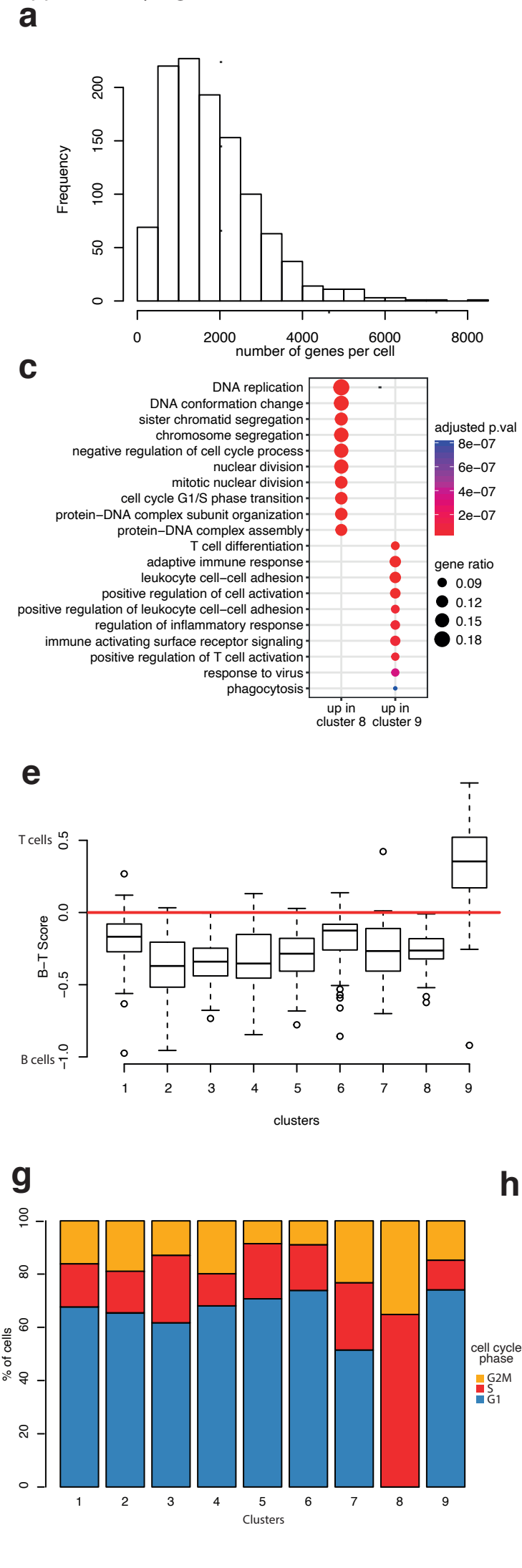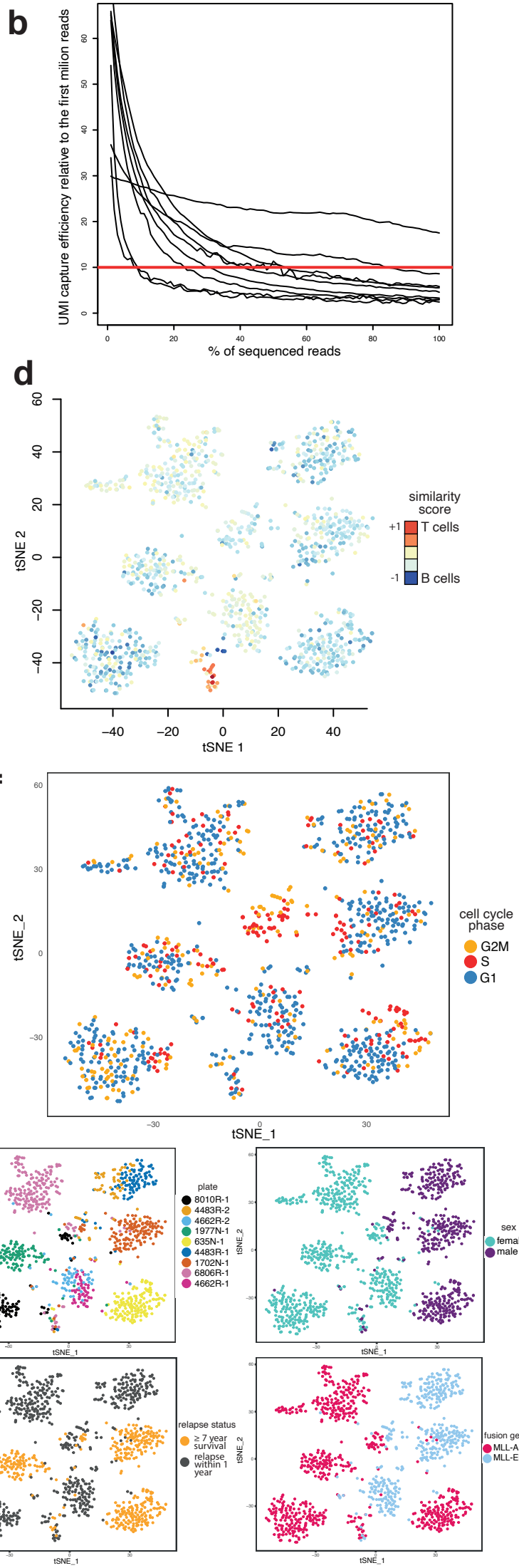

Supplement: Supplementary file 7 — Supplementary Figure 2 [file 41375_2021_1341_MOESM7_ESM.pdf]

**a**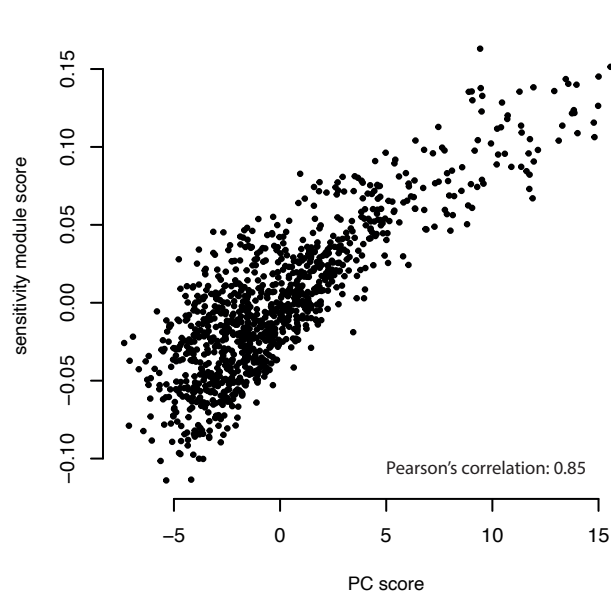**b**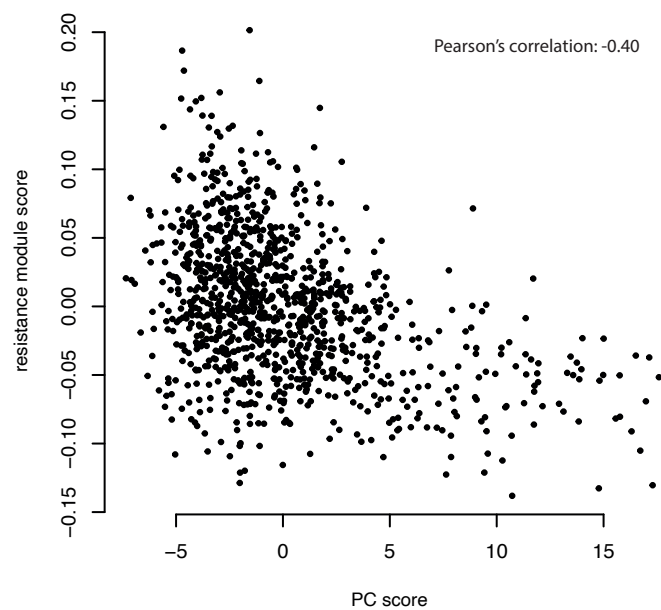**c**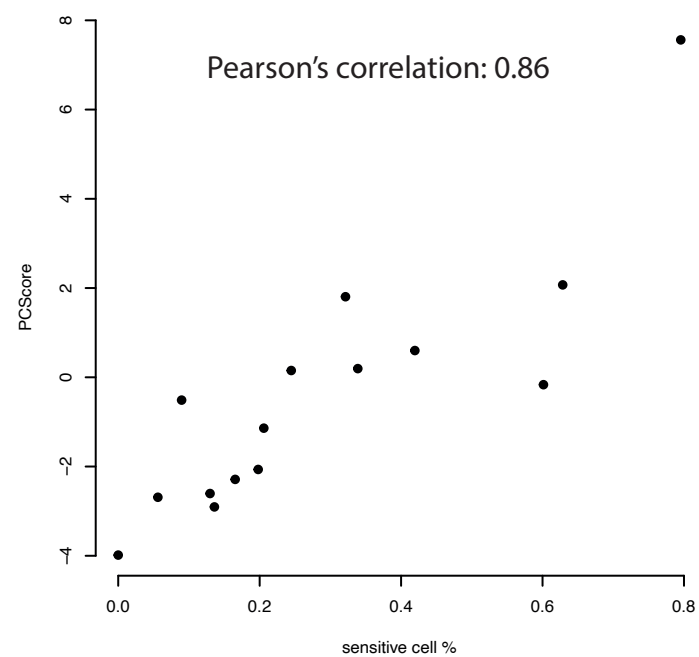**d**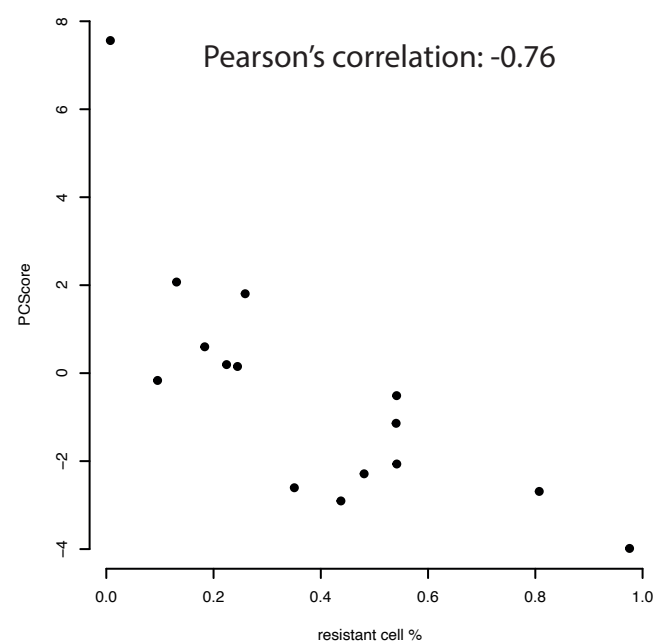

Supplement: Supplementary file 8 — Supplementary Figure 3 [file 41375_2021_1341_MOESM8_ESM.pdf]

**a**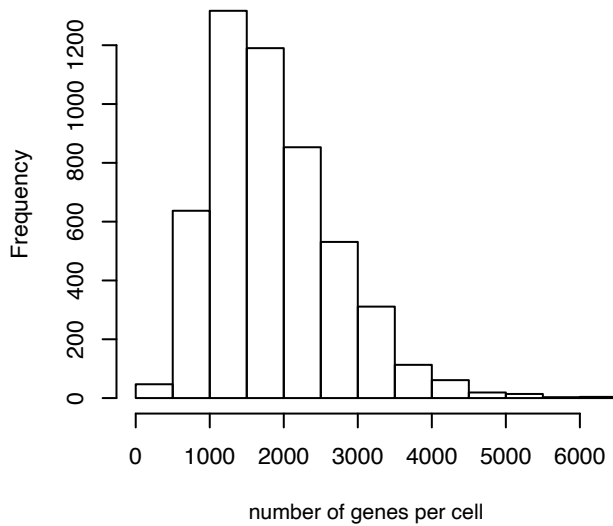**b**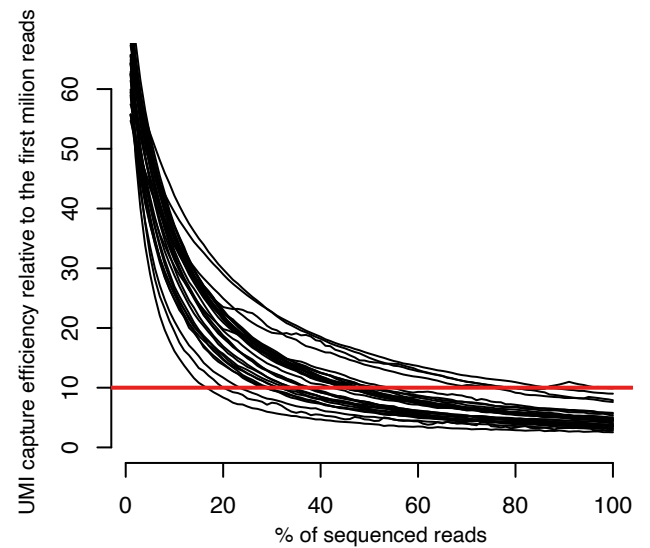**c**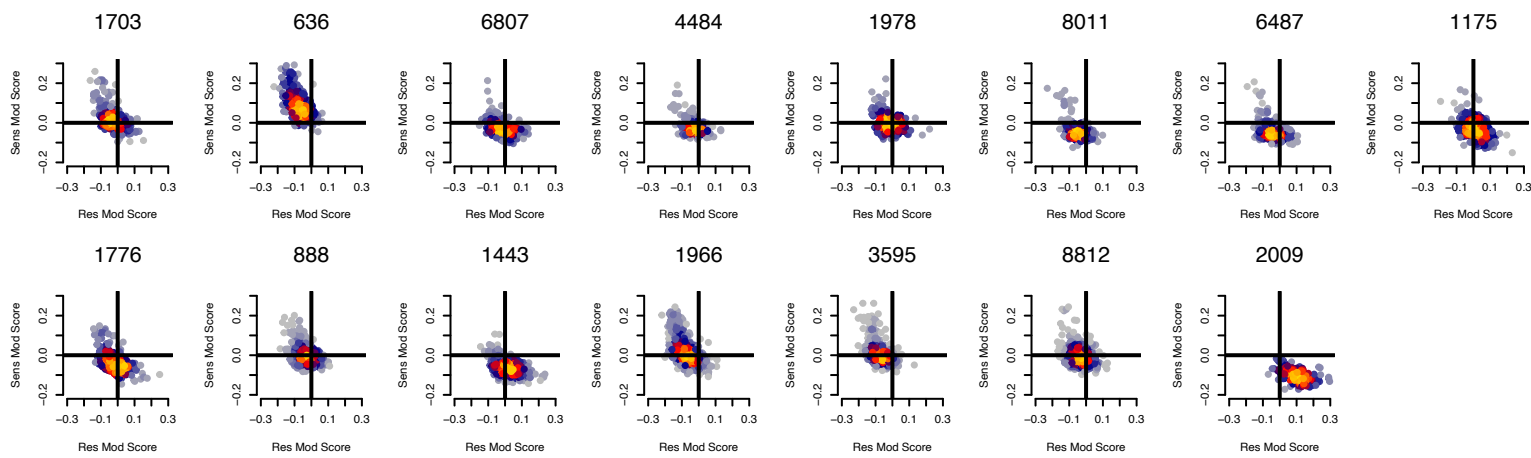**d**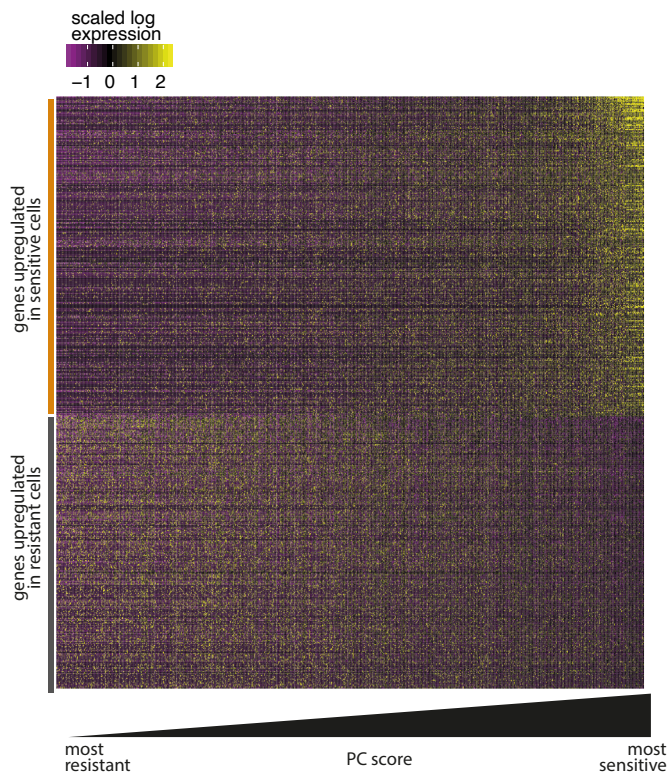**e**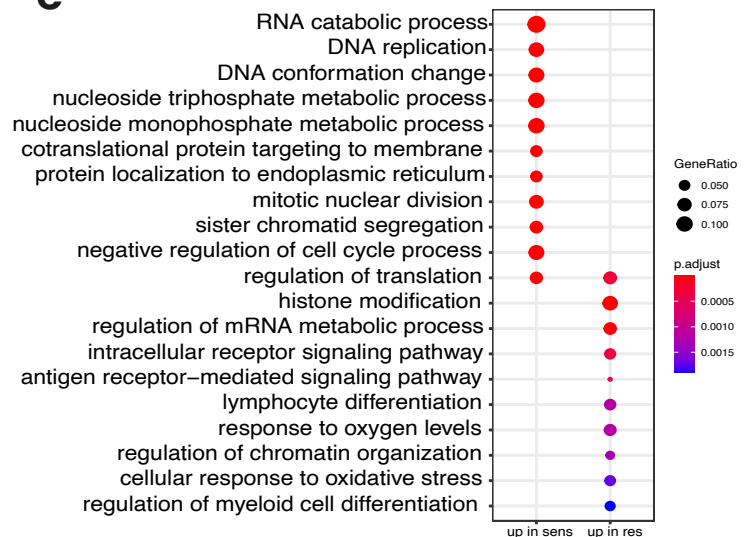**f**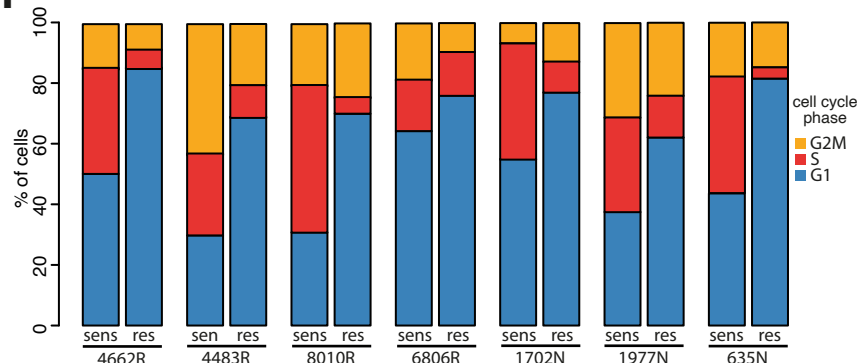

Supplement: Supplementary file 10 — Supplementary Figure 5 [file 41375_2021_1341_MOESM10_ESM.pdf]

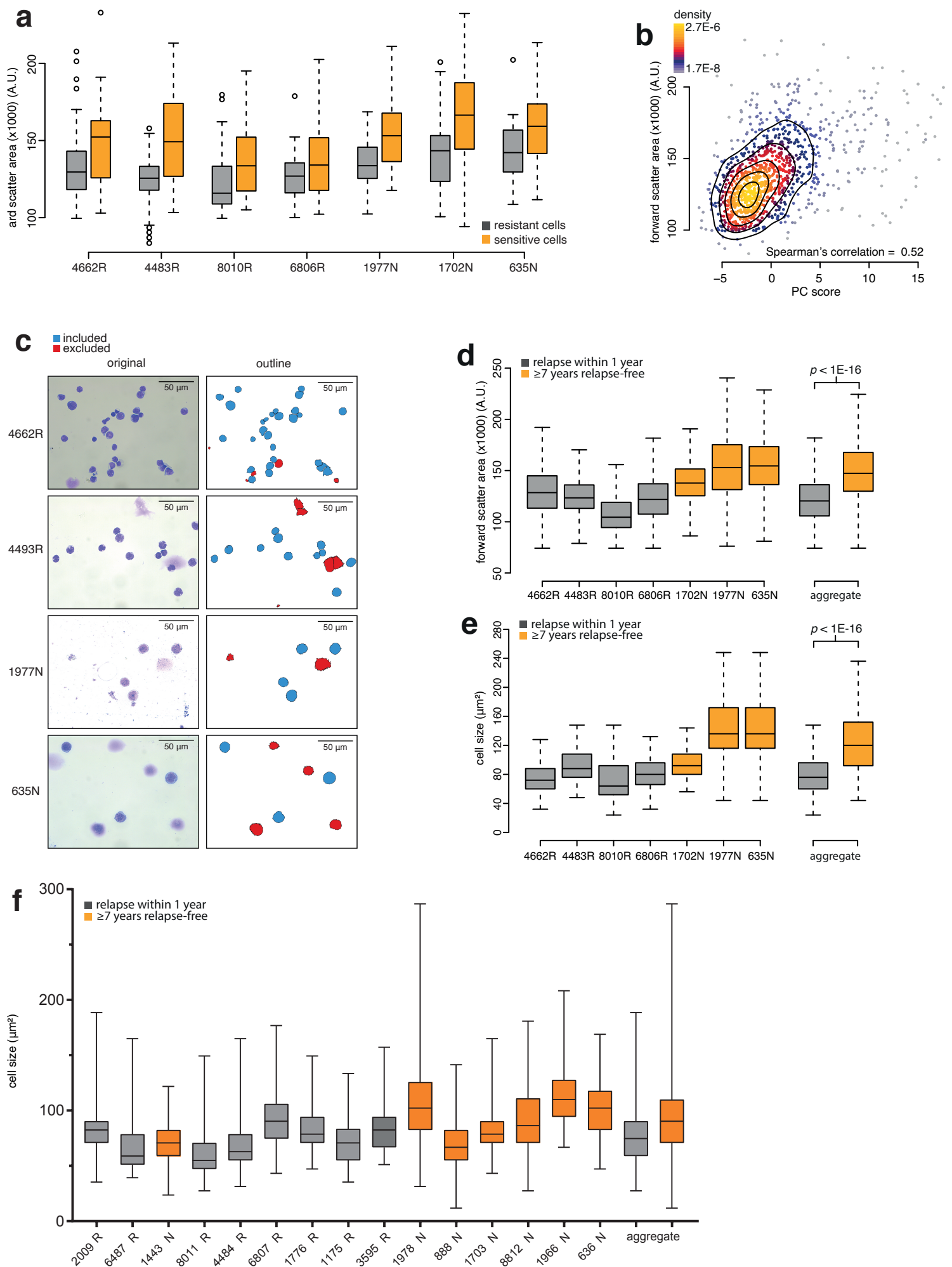

Supplement: Supplementary file 11 — Supplementary Figure 6 [file 41375_2021_1341_MOESM11_ESM.pdf]

Supplementary Figure 7

**a**

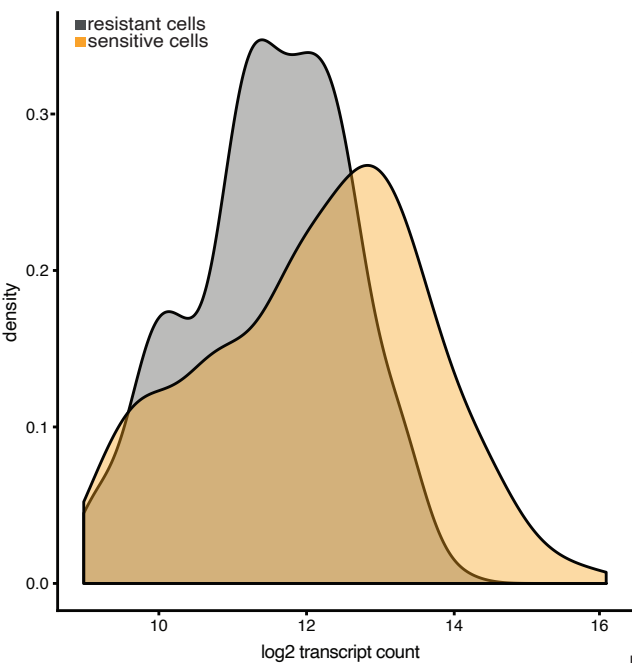

**b**

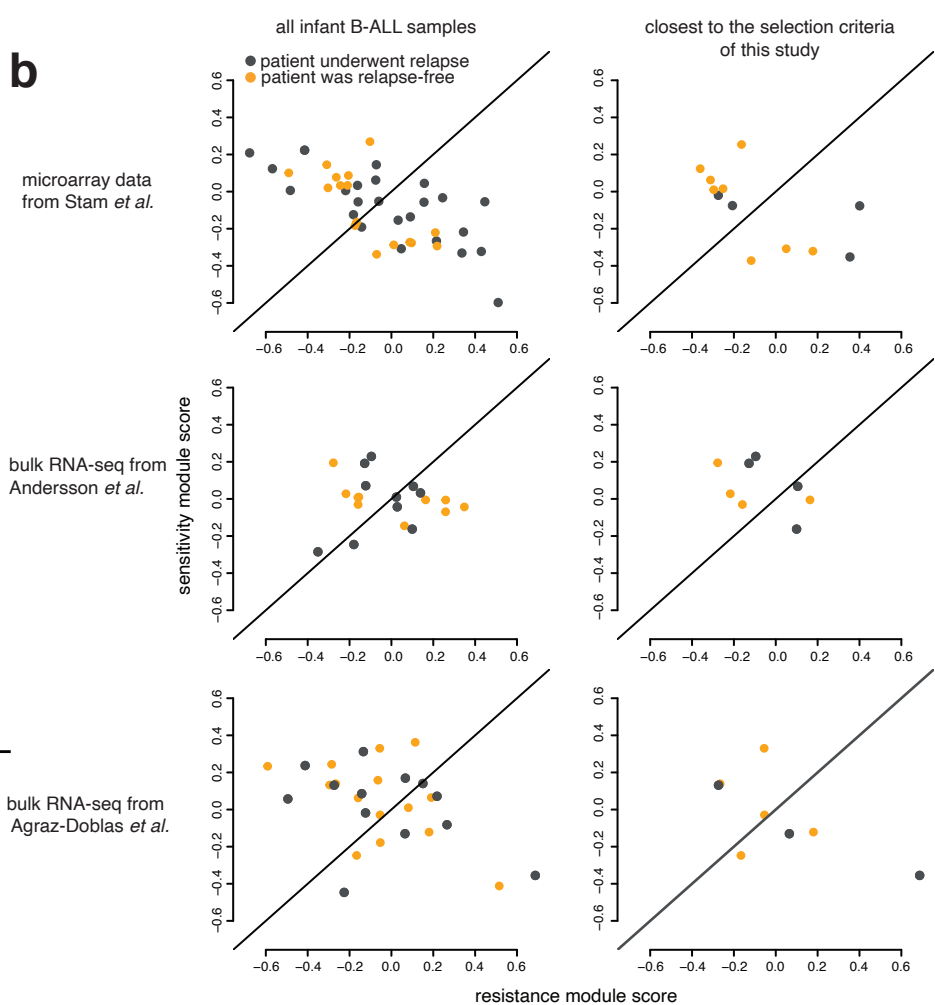

**c**

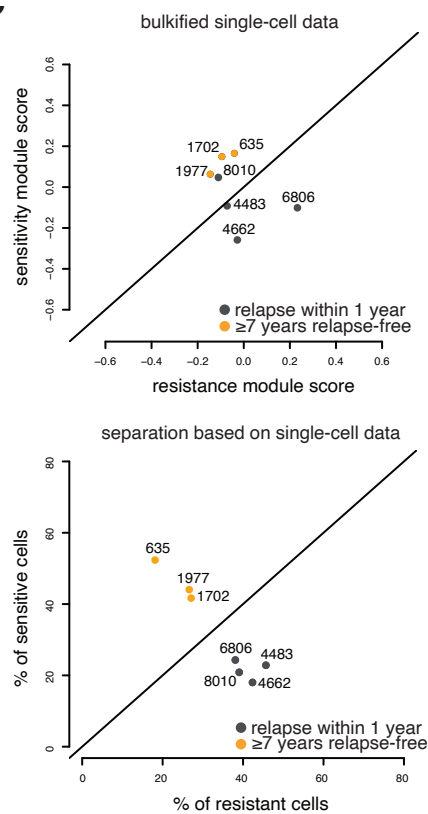

**d**

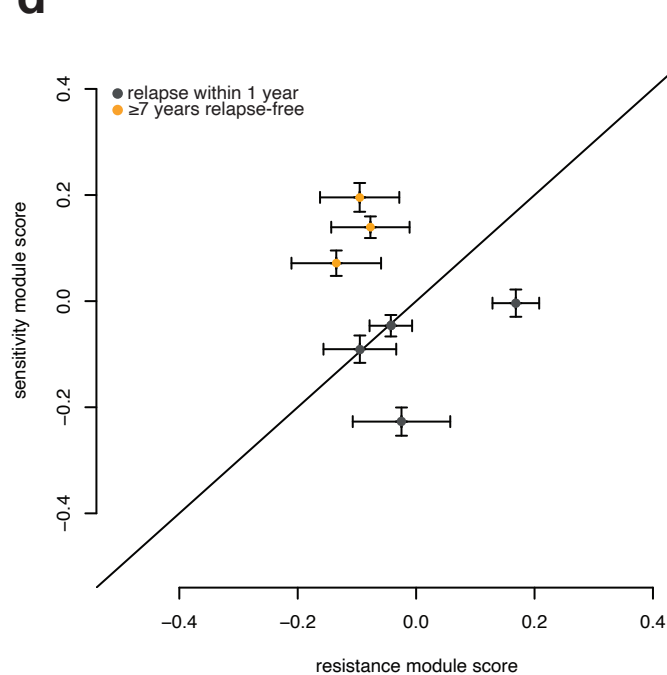

Supplement: Supplementary file 12 — Supplementary Figure 7 [file 41375_2021_1341_MOESM12_ESM.pdf]
