# Supplementary material for: Identification and characterization of relapse-initiating cells in MLL-rearranged infant ALL by single-cell transcriptomics
Source: Leukemia. 2021 Jul 24;36(1):58–67. doi: 10.1038/s41375-021-01341-y (PMC8727302; doi:10.1038/s41375-021-01341-y)

Supplementary Figure 4

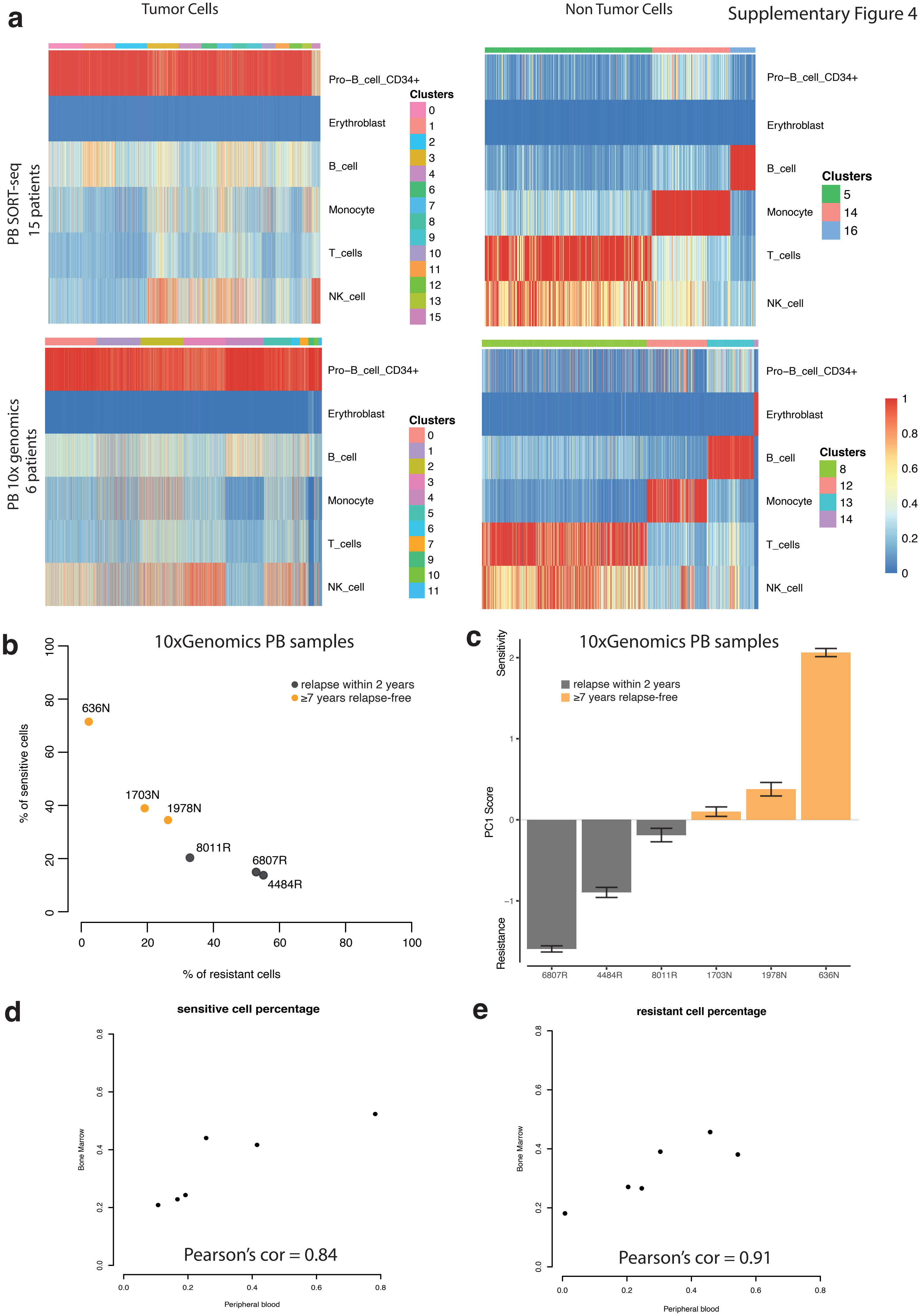

Supplement: Supplementary file 9 — Supplementary Figure 4 [file 41375_2021_1341_MOESM9_ESM.pdf]
