# Supplementary material for: Identification and characterization of relapse-initiating cells in MLL-rearranged infant ALL by single-cell transcriptomics
Source: Leukemia. 2021 Jul 24;36(1):58–67. doi: 10.1038/s41375-021-01341-y (PMC8727302; doi:10.1038/s41375-021-01341-y)
Supplement: Supplementary file 13 — Supplementary figure legends [file 41375_2021_1341_MOESM13_ESM.docx]

**Supplemental Figure 1: FACS gating strategy**

Gating strategy used to sort all samples described in this study. **a,** gating strategy for the FACSAria III **b,** gating strategy for the MoFlo Astrios

**Supplementary Figure 2: Leukemic cells group largely according to patient**

**a,** histogram showing the frequency of the number of genes detected per cell. **b,** UMI capture efficiency shown as a function of the percentage of sequenced reads (see sup methods). A final efficiency of 10 implies that only 10% of the total complexity of the dataset is left unsequenced. **c,** Gene Ontology enrichment based on cluster 8- and 9-specific genes indicates a high degree of actively dividing cells in cluster 8 and indicates that cluster 9 represents T-cells. **d,** Similarity score based on expression of T- and B-cell markers projected on a t-SNE plot. **e,** boxplot showing the distribution of the B-T similarity score for each cluster. **f,** Distribution of cell cycle phase on the t-SNE plot indicates a high degree of actively dividing cells in cluster 8 (fig 1c). **g**, For each cluster (Figure 1c), the fraction of cells assigned to the different cell cycle phases shows that cluster 8 consists entirely of cycling cells (G2M and S). **h**, t-SNE plots with cells labelled by FACS plate, sex, relapse status, or fusion partner indicate absence of batch effects.

**Supplementary Figure 3: Principal component versus gene module scores**

**a,** PC score correlation with the sensitivity module score and **b**, anti-correlation with the resistance module score in individual cells. This indicates that signals from both sensitivity and resistance modules are captured within the first PC and that the contribution from the sensitivity module is stronger, likely also because it had more genes. **c-d,** average PC score per patient correlated with the % of sensitive or resistant cells, respectively.

**Supplementary Figure 4: Sequencing of an expanded cohort of peripheral blood samples with SORT-seq and 10xGenomics.**

**a,** SingleR heatmap showing correlations between cells (columns) and cell types (rows) for peripheral blood samples. Cells are split by tumor or non-tumor and processed with SORT-seq or 10xGenomics. **b,** quantitation of the proportion of sensitive and resistant cells in six PB samples processed with 10xGenomics. **c,** PC score calculated on the PB samples processed with 10xGenomics. **d-e,** correlation of sensitive and resistant cell percentage between BM and PB samples from the same patients.

**Supplementary Figure 5**: **Characterization of sensitive and resistant cells in bone marrow samples**

**a,** Frequency distribution of genes per cell in all SORT-seq processed PB samples. **b,** UMI capture efficiency shown as a function of the percentage of sequenced reads (see sup methods). A final efficiency of 10 implies that only 10% of the total complexity of the dataset is left unsequenced. **c,** resistance and sensitivity module scores (x- and y-axis) plotted over all cells for each patient’s SORT-seq PB sample. **d,** Expression heatmap of all differentially expressed genes between cells classified as sensitive and resistant. cells (columns) are ordered by PC score, reflecting a gradient from resistant to sensitive. **e**, Gene Ontology categories enriched in the upregulated genes in sensitive and resistant cells. Gene ratio represents the fraction of differentially expressed genes in each category **f**, Cell cycle analysis of cells predicted to be sensitive or resistant in the different samples.

**Supplementary Figure 6**: **evaluation of size as an indicator of sensitivity/resistance.**

**a,** Boxplots showing the distribution of FACS forward scatter area values for cells classified as sentitive and resistant in individual bone marrow samples. **b**, Correlation between FACS forward scatter area and the first PC score of individual bone marrow cells analysed by scRNA-seq. **c,** Examples of Cytospin images and their digitally outlined counterparts with excluded objects in red, as used for cell size analysis. **d**, Distribution of forward scatter area values of all cells from individual bone marrow samples shown as box plots. Each sample’s contribution to the aggregates is equal. **e**, Distribution of cell sizes as detected by microscopy of all cells from individual bone marrow samples shown as boxplots. Each sample’s contribution to the aggregates is equal. **f**, Distribution of cell sizes as detected by microscopy of all cells from individual peripheral blood samples shown as boxplots. Each sample’s contribution to the aggregates is equal

**Supplemental Figure 7: Lower amounts of transcripts in relapse-associated cells hampers classification through bulk expression analyses**
**a,** Distribution of transcript counts for cells classified as sensitive or resistant. Resistant cells have approximately half as many transcripts as sensitive cells (0.52 ratio, p = 8E-10). Note the log2 scale and the skewed distribution of high transcript abundance in sensitive cells. **b,** Sensitivity and resistance module score plots for three bulk datasets. Plots on the left include all infants with B-cell ALL in each study while plots on the right include only patients that are closest to our bone marrow study cohort (bone marrow samples, t(4;11) and t(11;19), relapse within 1 year or at least 7 years relapse-free survival. **c,** Sensitivity/resistance module score plot calculated for bulkified single cell RNA-sequencing

datasets (top) compared with classification based on single cells (bottom, identical to Figure 1g). **d,** Sensitivity/resistance module score plot calculated for bulkified single-cell RNA- sequencing datasets where each cell contributes equally to the dataset. Error bars represent the standard error of the mean from 30 samplings.
